# Analysis of risk factors for axial symptoms after posterior cervical open-door laminoplasty

**DOI:** 10.1186/s13018-023-04426-9

**Published:** 2023-12-11

**Authors:** Chaoyue Ruan, Weiyu Jiang, Wenjie Lu, Yang Wang, Xudong Hu, Weihu Ma

**Affiliations:** Department of Spinal Surgery, Ningbo Sixth Hospital, Ningbo, 315040 Zhejiang China

**Keywords:** Cervical spondylotic myelopathy, Laminoplasty, Axial symptoms, Risk factors, Cutoff value

## Abstract

**Background:**

Laminoplasty (LP), a procedure commonly used to treat cervical spondylotic myelopathy (CSM), often results in the development of axial symptoms (AS) postoperatively. This study aims to analyze the risk factors associated with the occurrence of AS after LP.

**Methods:**

We collected and evaluated clinical data from 264 patients with CSM who underwent LP treatment at our institution from January 2018 to January 2022 through a single-center retrospective study. Of the patients, 153 were male and 111 were female, with an average age of 58.1 ± 6.7 years. All patients underwent C3-7 posterior laminoplasty. Based on the occurrence of postoperative axial symptoms, the patients were divided into an AS group and a non-AS group. General information, including age, gender, disease duration, Japanese Orthopaedic Association (JOA) score, postoperation early function training, and collar-wearing time, was recorded and compared between the two groups. Surgical-related data, such as operative segments, surgical time, intraoperative blood loss, intraoperative facet joint destruction, and destruction of the C7 spinous process muscle insertion, were also compared. Imaging data, including preoperative cervical curvature, cervical range of motion, preoperative encroachment rate of the anterior spinal canal, and angle of laminar opening, were collected. Univariate and multivariate logistic regression analyses were used to identify risk factors for the development of AS after LP, and receiver operator characteristic (ROC) curves were utilized to explore the optimal preoperative parameters.

**Results:**

All 264 patients successfully underwent surgery and were followed up for an average of 19.5 ± 6.8 months. At the 6-month follow-up, 117 patients were diagnosed with AS, resulting in an incidence rate of 40.2%. The multivariate logistic regression analysis identified that preoperative encroachment rate of anterior spinal canal (Pre-op ERASC), intraoperative facet joints destruction (Intra-op FJD), intraoperative open-door angle (Intra-op OA), destroy the C7 spinous process muscle insertion (Destroy C7 SPMI), postoperative loss of cervical curvature (Post-op LCC), and postoperative loss of cervical range of motion (Post-op LCROM) were independent risk factors for AS. Conversely, preoperative cervical curvature (Pre-op CC) and postoperation early function training (Post-op EFT) were protective factors against AS. According to the ROC curve, the cutoff values for preoperative anterior spinal canal occupation rate and preoperative cervical curvature were 28.5% and 16.5°, respectively. When the preoperative anterior spinal canal occupation rate was greater than 28.5% or the preoperative cervical curvature was less than 16.5°, AS was more likely to occur after surgery.

**Conclusion:**

High preoperative anterior spinal canal occupation rate, facet joint damage during surgery, C7 spinous process muscle stop point damage, larger angle of laminar opening, and greater postoperative cervical curvature loss and cervical range of motion loss are associated with an increased risk of developing AS after cervical laminoplasty. Conversely, a larger preoperative cervical curvature and early postoperative functional exercises can help reduce the occurrence of AS.

## Background

Cervical spondylotic myelopathy (CSM) is a clinically common and severe disease with a prolonged course and progressive development. It is often caused by cervical disk protrusion or facet joint degeneration, leading to spinal cord compression and ischemia. Once diagnosed, early surgical treatment is recommended for patients [1, 2]. Laminoplasty (LP) is a commonly used surgical technique for treating CSM. During the procedure, the posterior vertebral lamina is opened at a specific angle to increase the volume of the spinal canal. This allows the spinal cord to drift backward under the “bowstring effect,” avoiding anterior compression and alleviating spinal cord compression. Compared to anterior cervical decompression and fusion surgery, LP offers advantages such as a larger surgical field, shorter operative time, simpler procedure, less impact on cervical spine mobility, and the ability to address multilevel spinal cord compression simultaneously [3–6]. However, LP involves extensive dissection of the posterior cervical muscle tissue, removal of spinous processes and ligaments, and alteration of the original anatomical structures, which may result in complications such as C5 nerve root paralysis, biomechanical imbalance, and axial symptoms, with an AS incidence ranging from 37 to 80% [7–9]. Despite achieving favorable neurological recovery postoperatively, persistent neck and shoulder pain can significantly affect patients' daily life and work. Recent research has indicated that the occurrence of AS is related to the destruction of the posterior cervical muscle-ligament complex, decreased cervical mobility, facet joint degeneration, and prolonged collar-wearing, but the clinical data quality of these studies varies, and some findings even contradict each other. As a result, there is currently no unified understanding of the independent risk factors for developing AS after LP [10–12]. Therefore, this study aims to analyze the clinical data of 264 CSM patients who underwent LP treatment at our institution from January 2018 to January 2022, in combination with relevant previous research. Through univariate analysis and multivariate logistic regression, we aim to identify the risk factors for AS occurrence after LP and explore the optimal preoperative parameters using the receiver operator characteristic curve and Youden index. The findings will guide preoperative preparation, intraoperative procedures, and postoperative care to reduce the incidence of AS.

### Evidence before this study

We searched PubMed, Medline, and CSTJ for peer-reviewed, original studies published from database inception to January 2022, with the terms “laminoplasty,” “LP,” “axial symptoms,” AS, and “risk factors.” It is hoped that this study can include enough risk factors in order to provide some ideas for the prevention and treatment of axial symptoms [7, 13, 14].

## Materials and methods

Inclusion and Exclusion Criteria: Inclusion criteria were as follows: (1) Patients diagnosed with CSM; (2) Imaging studies (cervical X-ray, CT, MRI) indicating compression of three or more spinal segments, with or without developmental cervical canal stenosis; (3) Surgical treatment with C3-7 posterior laminoplasty; (4) Follow-up period of at least 12 months with complete follow-up data. Exclusion criteria were as follows: (1) Patients with cervical spine trauma, fractures, or tumors; (2) Patients with preoperative cervical kyphosis requiring concurrent posterior internal fixation or anterior cervical surgery; (3) Patients who underwent combined anterior and posterior approach surgery or revision surgery; (4) Patients with poor compliance or unable to complete the follow-up as scheduled.

### Demographic and case grouping

A retrospective analysis was conducted on clinical data of patients with CSM treated at our institution from January 2018 to January 2022. A total of 264 patients were included in this study, comprising 153 males and 111 females, with an average age of 58.1 ± 6.7 years. All patients underwent C3-7 posterior laminoplasty. At the 6-month follow-up, we used the diagnostic criteria for axial symptoms proposed by Takeuchi et al. [15], which included the following characteristics: (1) Severe pain with minimal movement; (2) Absence of local tenderness; (3) Pain relief with warmth and exacerbation with cold; (4) Pain relief while lying flat. If these symptoms persisted for more than 1 month, patients were diagnosed with cervical axial symptoms. Based on the occurrence of axial symptoms, the patients were divided into two groups: the AS group (106 cases; 62 males, 44 females; average age of 55.1 ± 9.2 years) and the non-AS group (158 cases; 91 males, 67 females; average age of 52.4 ± 11.6 years).

### Surgical procedure and postoperative management

Laminoplasty was performed by two experienced surgeons (W-Y.J. and W-H.M.) from the same institution. After the induction of general anesthesia, the patient is placed in a prone position, and a midline incision is made at the posterior aspect of the neck. The C4–7 bilateral spinous processes, laminae, and partial facet joints are exposed by preserving the muscles attached to the C2–3 spinous processes. The interspinous ligaments between C3–4 and C7–T1 are then severed and bitten away, revealing the ligamentum flavum. Utilizing two towel clamps, the spinous process base of C3–4 is clamped and pulled upward and downward, respectively, to widen the interlaminar space. At this point, a clear view of the inferior border of the C3 vertebral body is obtained. Following the dissection and separation of the ligamentum flavum attached to the inferior border of the C3 vertebral body, a thin laminar bone rongeur is inserted parallel to the interlaminar space. After rotating 90° against the vertebral body, the rongeur is advanced to nibble away half of the inferior border of the C3 vertebral body and the root of the spinous process, thereby disconnecting the ligamentum flavum between C3 and C4 and clearly exposing the dura mater. Similarly, towel clamps are applied to the spinous process bases of C7 and T1 to enlarge the interlaminar space and expose the ligamentum flavum. The upper half of the T1 lamina is then removed with a laminar bone rongeur. At the C4–7 hinge side of the lamina groove, the outer cortical bone is bitten away with a microcurved, pointed bone rongeur. By combining the microcurved bone rongeur with a thin laminar bone rongeur, the outer edge of the lamina is bitten off on the open side of the lamina groove. The lamina is then lifted, and a micro-titanium plate is used to separate and fix the open-side lamina and facet joint, maintaining the lamina in an open position. Hemostasis is carefully achieved postoperatively, and the wound is irrigated before the placement of a drainage tube. The incision is closed layer by layer, covered with a dressing, and immobilized. The surgical procedure concludes at this point.

Postoperative care included routine symptomatic treatment such as infection prevention, anti-inflammatory measures, and edema reduction. Patients were encouraged to perform active limb movements while in bed. The drainage tube was removed based on the drainage situation. Three days after surgery, patients were allowed to perform appropriate activities with neck protection. One week post-surgery, cervical X-rays and CT scans were performed in the neutral and dynamic positions to assess the surgical outcome. Two weeks after the operation, neck muscle exercise was performed early according to the condition of each patient to prevent the atrophy of the posterior cervical extensor muscle. The neck brace was worn for 6–8 weeks. The outpatient clinic was reviewed regularly at 3, 6, and 12 months after the operation. Nonsteroidal anti-inflammatory drugs and rehabilitation physiotherapy exercises were instructed to prevent axial symptoms when discharged.

## Evaluation indicators

### General information

Data on age, gender, disease duration, JOA score, JOA improvement rate, early postoperative functional exercises, and collar-wearing time were recorded and compared between the two groups. The JOA score ranges from 0 to 17 points, and the JOA improvement rate is calculated as (postoperative JOA score-preoperative JOA score)/(17-preoperative JOA score) × 100%. Information on early postoperative functional exercises and collar-wearing time was obtained through telephone follow-up.

### Surgical-related data

Data on surgical segments, surgical time, intraoperative blood loss, and occurrences of facet joint damage and C7 spinous process muscle stop point damage were recorded and compared between the two groups.

### Imaging data

Imaging data include cervical curvature, cervical range of motion, preoperative anterior spinal canal occupation rate, and angle of laminar opening.

#### Cervical curvature and cervical range of motion

**Fig. 1:**
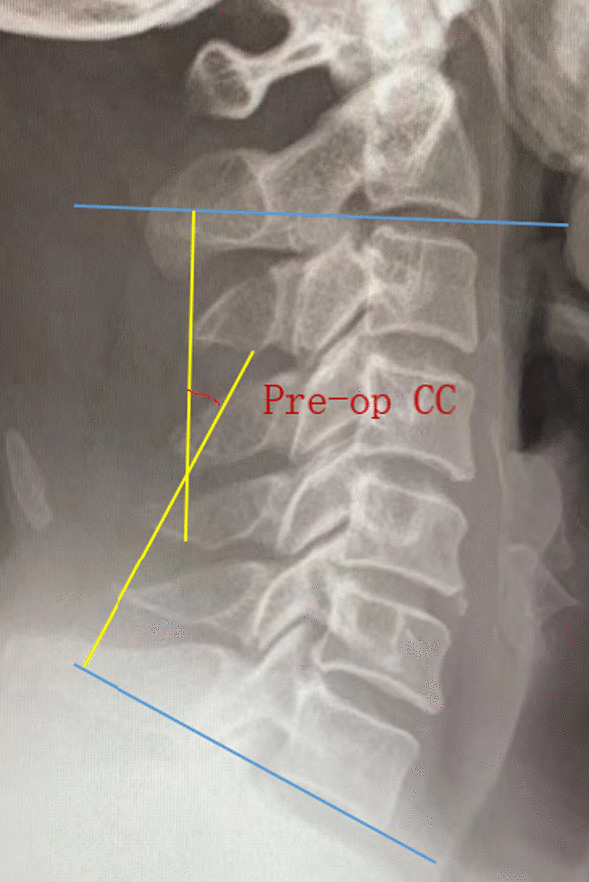
The cervical spine was taken in a neutral position, and the cervical curvature was represented by the angle between the tangent line of C2 and the lower edge of C7 vertebral body. The loss of cervical curvature = postoperative cervical curvature—preoperative cervical curvature

**Fig. 2:**
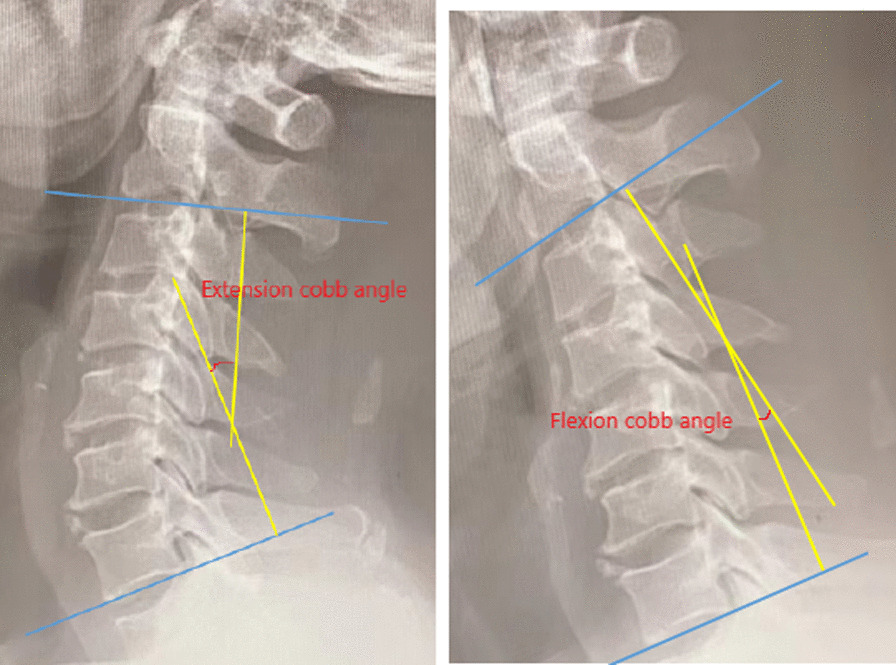
The hyperextension and hyperflexion position of cervical spine was taken before and after operation. Cervical range of motion = extension Cobb angle + flexion Cobb angle, loss of cervical range of motion = postoperative range of motion-preoperative range of motion

Cervical X-rays in neutral and dynamic positions were performed before surgery and one week after surgery. The angle formed by the lines drawn along the lower edges of the C2 and C7 vertebral bodies represents cervical curvature (Fig. 1). Cervical curvature loss is calculated as postoperative cervical curvature—preoperative cervical curvature. Cervical range of motion is represented by the sum of C2–C7 Cobb angles in cervical extension and flexion positions (Fig. 2). Cervical range of motion loss is calculated as postoperative range of motion—preoperative range of motion.

#### Preoperative encroachment rate of anterior spinal canal

**Fig. 3:**
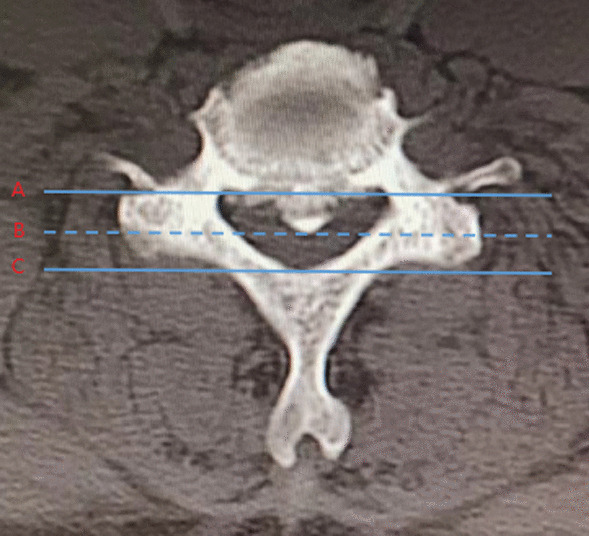
Preoperative encroachment rate of anterior spinal canal = AB / AC × 100%

The preoperative encroachment rate of anterior spinal canal is determined based on the horizontal T2-weighted MRI image of the most severely compressed cervical segment before surgery. Three horizontal lines (A, B, C) are drawn on this image, representing the anterior margin of the spinal canal, the level of anterior spinal cord compression, and the posterior margin of the spinal canal, respectively (Fig. 3). The anterior spinal canal occupation rate is calculated as AB/AC × 100%.

#### Opening angle

**Fig. 4:**
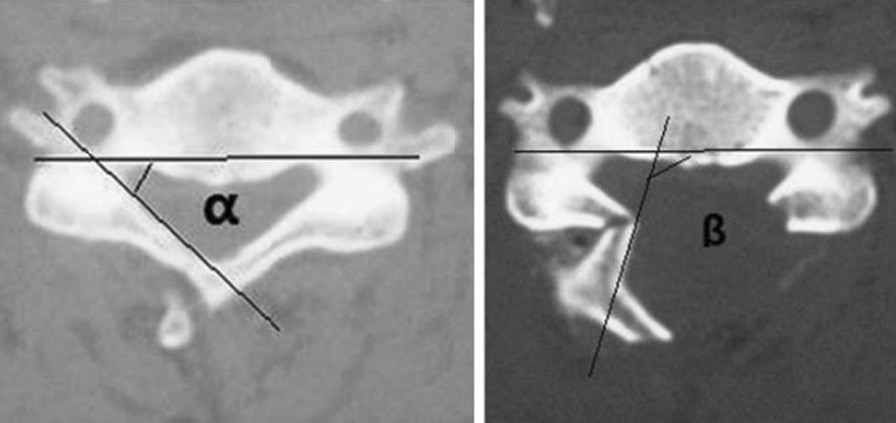
Opening angle = postoperative lamina angle (*β*)-preoperative lamina angle (*α*). The average value of the opening angle of each segment is the intraoperative opening angle

The laminar opening angle is measured on CT scans before surgery and one week after surgery, using the method described by Zhang et al. [16]. The laminar opening angle is defined as the angle formed by two intersecting lines: one line passing through the posterior wall of the bilateral transverse foramen of the vertebral body, and the other line is the inner edge tangent of the lateral mass on the hinge side. The laminar opening angle is calculated as the postoperative laminar angle (*β*) minus the preoperative laminar angle (*α*) (Fig. 4). The average value of the measured laminar opening angles at each segment represents the intraoperative laminar opening angle.

The data mentioned above were measured by two experienced professionals: one spinal surgeon with over 10 years of experience in spine surgery and one radiologist specialized in the musculoskeletal system. Both experts independently measured the data, and the final results were obtained by calculating the average of the two measurements from each professional to ensure accuracy and reduce measurement bias.

## Statistical methods

All data were statistically analyzed using SPSS 25.0 software. Normally distributed continuous data are presented as mean ± standard deviation (*X* ± *S*) and were analyzed using independent sample t tests. Non-normally distributed continuous data are presented as median (minimum–maximum) and were analyzed using the Mann–Whitney *U* test. Categorical data were analyzed using the Chi-square (*X*^2^) test. One-way analysis of variance (ANOVA) was used to compare the mean differences of various indicators at different time points, and post hoc comparisons were performed using the least significant difference (LSD) method. Univariate analysis was conducted to identify significant variables, and multiple-factor logistic regression analysis was performed to identify risk factors for postoperative AS after LP surgery. ROC curve analysis was used to explore the cutoff value of preoperative parameters. A significance level of *P* < 0.05 was considered statistically significant.

## Results

Finally, a total of 264 patients were included in this study, and all patients were followed up for 19.5 ± 6.8 months. At the 6-month follow-up, 106 patients were included in the AS group and 158 patients were included in the non-AS group. The incidence of AS was 40.2% (106/264). Patients in the AS group failed to achieve satisfactory results after drug treatment and rehabilitation physiotherapy. During the follow-up period, no cases of restenosis were observed in either group, and there were no significant nerve or vascular injuries. One patient in each group experienced delayed wound healing postoperatively, but with appropriate wound care and dressing changes, the wounds eventually healed successfully. Moreover, there were no instances of wound infection reported during the follow-up period, and no cases of swan neck deformity were observed at the last follow-up.

### Univariate analysis of AS occurrence

The general characteristics of the two patient groups were compared, and statistically significant differences were observed in age, gender, early postoperative functional exercise, and duration of wearing a neck collar (*P* < 0.05). However, no statistically significant differences were found in the variables of disease duration, JOA score, and JOA improvement rate (*P* > 0.05). Regarding the surgical factors, there were no statistically significant differences between the two groups in terms of the surgical segments, surgical time, and intraoperative blood loss (*P* > 0.05). However, the occurrence of facet joint damage and C7 spinous process muscle endpoint damage was higher in the AS group than in the non-AS group, and these differences were statistically significant (*P* < 0.05). Regarding the imaging data, statistically significant differences were observed in preoperative cervical lordosis, loss of cervical lordosis, loss of cervical range of motion, anterior spinal canal occupation rate, and laminar opening angle (*P* < 0.05). However, no statistically significant differences were found in the remaining imaging parameters (*P* > 0.05) (Table 1).

**Table 1:** Single-factor analysis of AS after posterior cervical open-door laminoplasty

Indicators/groups	AS group (*n* = 106)	Non-AS group (*n* = 158)	Statistic	*P*-value
Age (years)	55.1 ± 9.2	52.4 ± 11.6	*t* = 2.009	0.046
Gender (male/female)	66/40	78/80	*x*^2^ = 4.256	0.039
Course of disease (months)	22.8 ± 20.1	19.7 ± 23.0	*t* = 1.128	0.260
Preoperative JOA score (points)	9.8 ± 3.3	10.2 ± 3.8	*t* = −0.883	0.378
Postoperative JOA score (points)	14.6 ± 2.7	14.1 ± 3.3	*t* = 1.296	0.196
JOA improvement rate (%)	51.9 ± 21.4	56.5 ± 24.3	*t* = −1.581	0.115
Wearing collar time (months)	2.7 ± 0.9	2.5 ± 0.7	*t* = 2.026	0.044
Postoperation early function training (examples)	31(29.2%)	78(49.4%)	*x*^2^ = 8.657	0.003
Surgical segments (pieces)	3.7 ± 0.5	3.6 ± 0.5	*t* = 1.593	0.112
Surgical time (min)	147.9 ± 33.5	139.1 ± 46.3	*t* = 1.683	0.094
Intraoperative bleeding volume (ml)	384.5 ± 72.6	371.9 ± 63.8	*t* = 1.488	0.138
intraoperative facet joints destruction (pieces)	1.6 ± 0.8	1.3 ± 0.5	*t* = 3.749	< 0.001
Destroy the C7 spinous process muscle insertion (cases)	18(17.0%)	13(8.2%)	*x*^2^ = 4.690	0.030
Preoperative cervical curvature(°)	17.1 ± 6.7	18.9 ± 6.4	*t* = −2.198	0.029
Postoperative cervical curvature (°)	12.6 ± 5.1	13.8 ± 6.3	*t* = −1.634	0.103
Loss of cervical curvature (°)	5.9 ± 4.3	4.6 ± 3.8	*t* = 2.583	0.010
Preoperative cervical range of motion (°)	43.2 ± 11.9	45.8 ± 14.7	*t* = −1.517	0.130
Postoperative cervical range of motion (°)	28.3 ± 12.6	31.5 ± 15.5	*t* = −1.769	0.078
Loss of cervical range of motion (°)	16.6 ± 9.3	13.2 ± 7.5	*t* = 3.275	0.001
Preoperative encroachment rate of anterior spinal canal (%)	28.8 ± 4.2	26.4 ± 3.7	*t* = 4.891	< 0.001
Opening angle (°)	40.7 ± 5.3	38.8 ± 5.3	*t* = 2.187	0.030

*P* < 0.05 is considered statistically significant

### Multivariable logistic regression analysis of AS occurrence

**Fig. 5:**
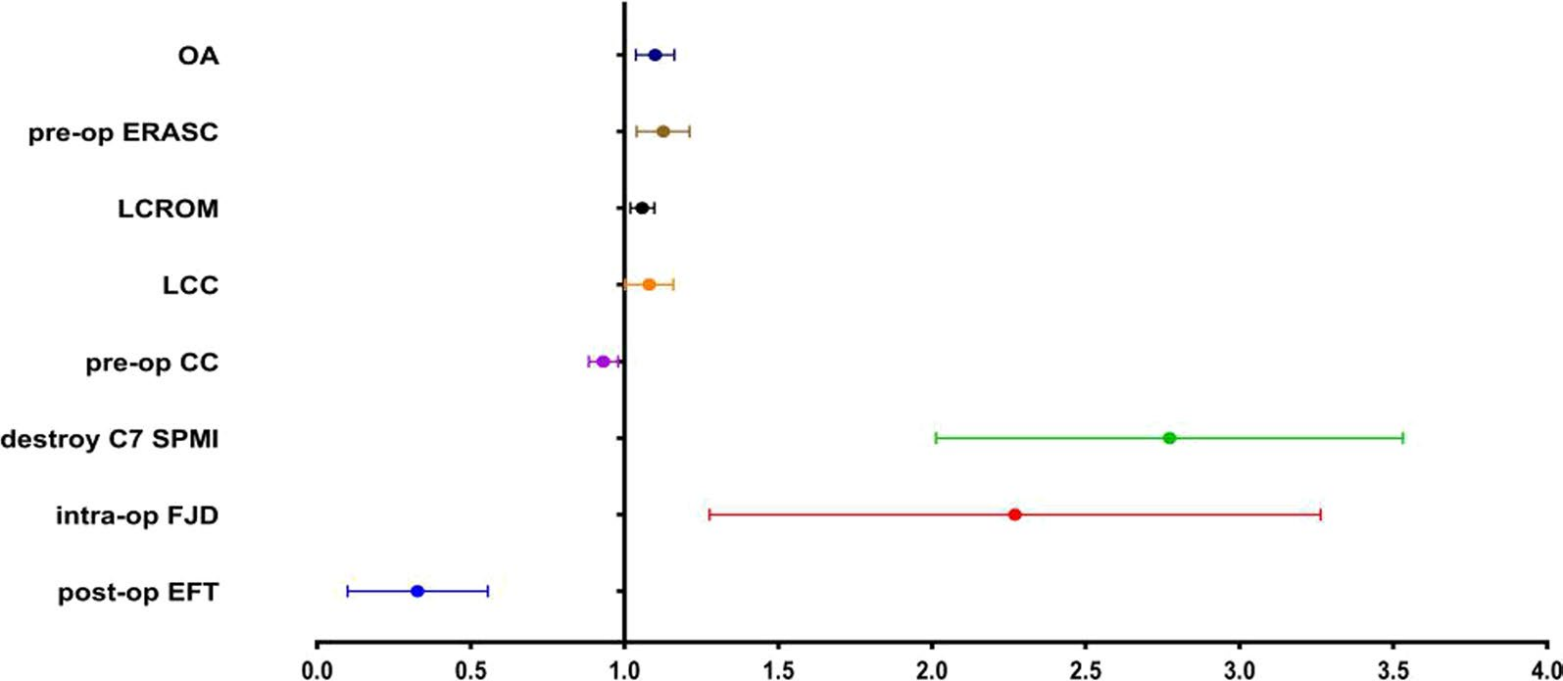
Forest plot of risk factors for AS after posterior cervical open-door laminoplasty. The OR for OA, Pre-op ERASC, LCROM, LCC, destroy C7 SPMI, and intra-op FJD are all less than 1, indicating that they are risk factors for the occurrence of AS after LP. Conversely, the OR values for Pre-op CC and Post-op EFT are both greater than 1, suggesting that they serve as protective factors against the development of AS following LP

The 11 variables that showed statistically significant differences in the univariate analysis were included in the multivariable logistic regression analysis using a backward stepwise method (Wald). The results revealed that high preoperative anterior spinal canal occupation rate (OR = 1.124, 95% CI 1.041–1.213), facet joint damage during surgery (OR = 2.125, 95% CI 1.357–3.328), C7 spinous process muscle endpoint damage (OR = 2.674, 95% CI 2.067–3.576), larger laminar opening angle (OR = 1.099, 95% CI 1.038–1.163), greater loss of cervical lordosis (OR = 1.078, 95% CI 1.002–1.160), and greater loss of cervical range of motion (OR = 1.124, 95% CI 1.041–1.213) were identified as independent risk factors for the occurrence of AS. On the other hand, higher preoperative cervical lordosis (OR = 0.931, 95% CI 0.885–0.979) and early postoperative functional exercise (OR = 0.274, 95% CI 0.130–0.577) were identified as protective factors against AS occurrence (Fig. 5).

### ROC curve analysis for AS prediction

**Fig. 6:**
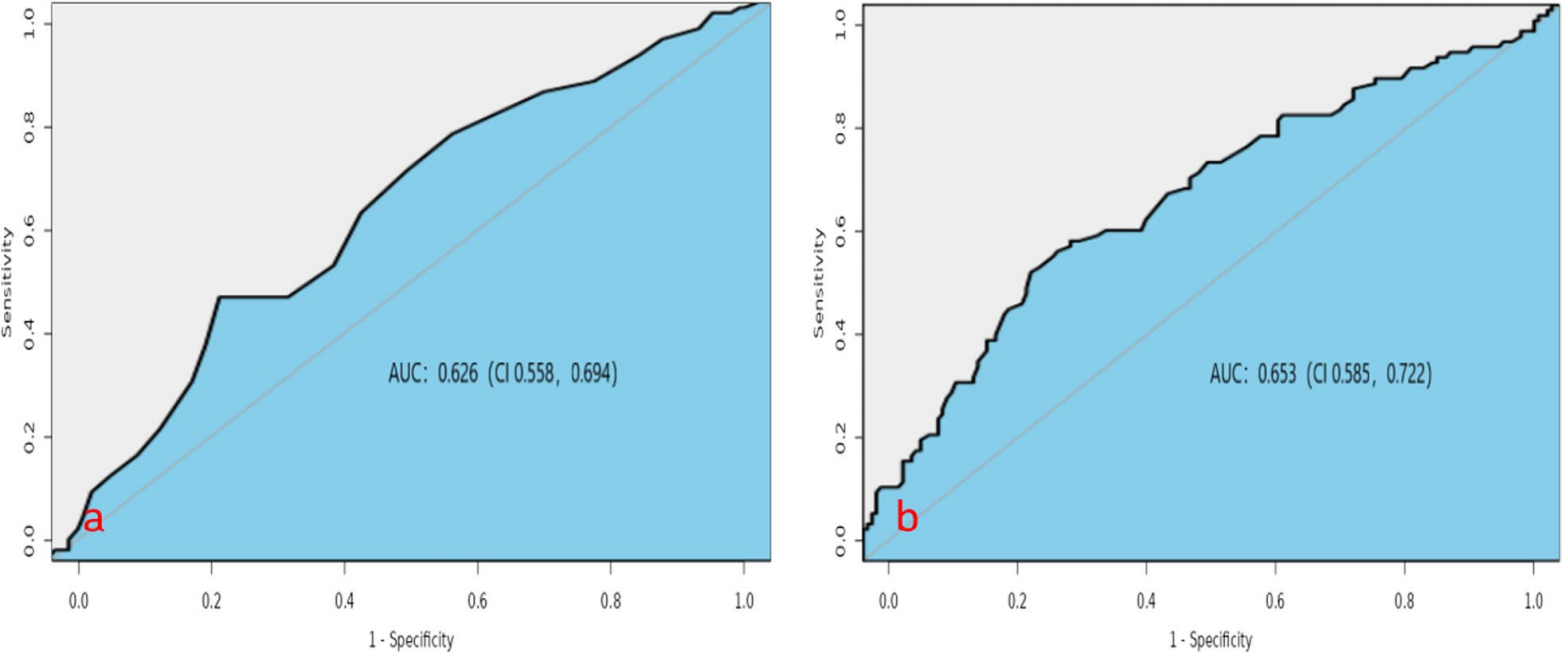
ROC curve of Pre-op CC, Pre-op ERASC, and AS risk. **a, b** The ROC area of Pre-op CC and Pre-op ERASC was 0.626 (CI 0.558, 0.694) and 0.653 (CI 0.585, 0.722), respectively, both greater than 0.5, suggesting that the predictive accuracy of these two risk factors for the occurrence of AS is general, considering that the occurrence of AS may be the result of a combination of multiple risk factors

**Fig. 7:**
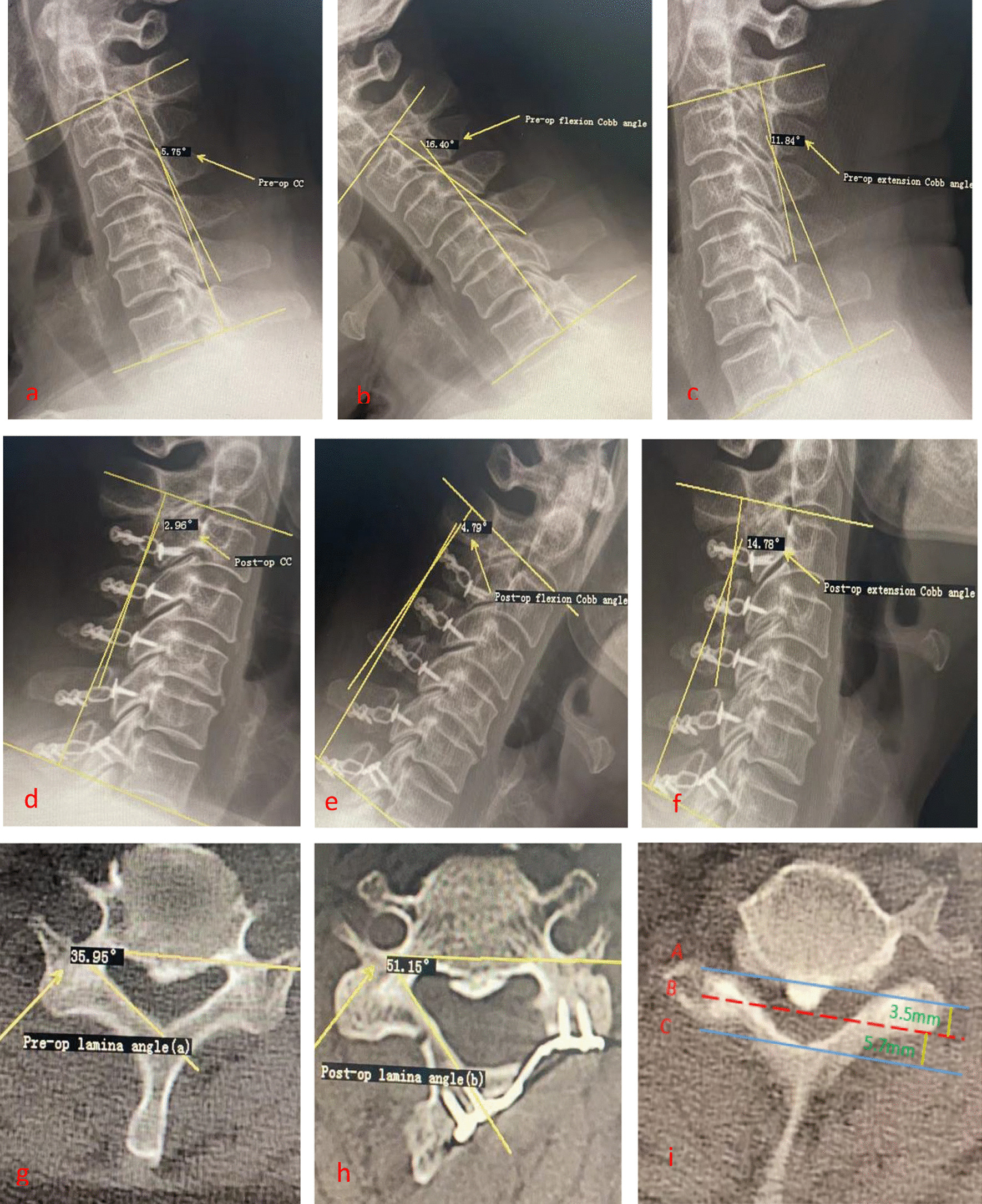
A 61-year-old female patient underwent C3-7 single-door posterior laminoplasty in our hospital. At 6 months after operation, the patient still had axial symptoms such as neck and shoulder pain and numbness. **a, d** The preoperative cervical curvature and postoperative cervical curvature were 5.8°and 3.0°, respectively, and the loss of cervical range of motion was 2.8°. **b, c, e, f** The preoperative cervical range of motion and postoperative cervical range of motion were 28.2°and 19.6°, respectively, and the loss of cervical range of motion was 8.6°. **g, h** The preoperative and postoperative lamina angles were 36.0°and 51.2°, respectively, and the opening angle was≈15.2°(only one segment was listed here, and the final opening angle was the average opening angle of all surgical stages, so ≈ was used here).** i** preoperative encroachment rate of anterior spinal canal = 38.0%

We performed ROC curve analysis with Pre-op CC and Pre-op ERASC as independent variables and the occurrence of AS as the dependent variable. The results showed that the maximum Youden's index for Pre-op CC was 0.238, corresponding to a cervical curvature value of 16.5° (sensitivity 0.766, specificity 0.472). This value can be considered as the cutoff point for Pre-op CC in diagnosing the occurrence of AS. Similarly, the maximum Youden's index for Pre-op ERASC was 0.279, corresponding to an occupation rate of 28.5% (sensitivity 0.577, specificity 0.722). This value can be used as the cutoff point for Pre-op ERASC in diagnosing the occurrence of AS (Fig. 6). A representative case is shown in Fig. 7.

## Representative case

### Discussion

Posterior cervical laminoplasty, as a common surgical procedure for treating myelopathic cervical spondylosis, not only demonstrates favorable clinical efficacy but also helps preserve cervical stability and motion function [17, 18]. However, one of the most prevalent complications after surgery is axial symptoms, which significantly impacts patients' quality of life [7–12, 14–16]. In this study of 264 patients, 106 cases developed AS postoperatively, resulting in an incidence rate of 40.2%, consistent with previous reports.

### Study on the pathogenesis of AS

The pathogenesis of AS remains uncertain, although it is widely accepted that multiple factors contribute to its occurrence [19–21]. Chen Chao et al. [22] suggested that axial symptoms may be related to damage to the posterior cervical ligamentous complex and small joint degeneration. Changes in cervical biomechanical properties after surgery disrupt normal biomechanical balance, leading to tension in damaged muscles, ligaments, and joint capsules, which stimulates pain receptors, ultimately causing clinical symptoms such as fatigue and pain. Z Liang et al. [23] found that preoperative inflammatory factors are often in an activated state in patients with axial symptoms, suggesting a lower inflammatory threshold. Severe damage to muscles, ligaments, and small joints during surgery may further activate inflammatory factors in peripheral nerve fibers, exacerbating the inflammatory response, and leading to pain and muscle spasms.

### Risk factors of AS

This study has revealed that the preoperative anterior encroachment rate on the spinal canal is a risk factor for the occurrence of AS, which aligns with the findings of Yusof and Naruse et al. [24, 25]. A higher preoperative anterior encroachment rate puts the spinal cord under prolonged compression, leading to potential damage and necrosis of the autonomic nerves that supply the blood vessels in the neck and shoulders. Consequently, this can result in symptoms such as neck and shoulder pain, numbness, and stiffness. Additionally, the limited space for posterior spinal cord movement may cause tension on connecting structures like the dentate ligament, leading to muscle fatigue and axial symptoms, including reduced sensation, pain, and limited mobility in the neck and shoulder regions. This study identified a cutoff value of 28.5% for the preoperative anterior encroachment rate, suggesting that when it exceeds 28.5%, there is a higher likelihood of AS development postoperatively. Thus, for patients with a preoperative anterior encroachment rate greater than 28.5%, postoperative rehabilitation therapy combined with neurotrophic medications may be considered for preventive treatment to reduce the incidence of AS.

The C7 spinous process plays a crucial role as an insertion point for the ligamentum nuchae, trapezius, and rhomboid muscles, contributing to the stability of the cervical spine. Previous studies, including Ono et al. [26] who conducted anatomical research on 50 cadavers, have confirmed that preserving the C7 spinous process effectively reduces muscle infiltration in the neck region. Lin W et al. [27], in a randomized single-blind controlled trial involving 96 patients, found that preserving the C7 spinous process better maintained cervical spine mobility and alignment in the sagittal plane, reduced blood loss, and lowered the incidence of axial symptoms. Hosono et al. [28] prospectively reduced the range of LP from C3–7 to C3–6, and found that the incidence of AS was significantly reduced after C3-6 laminoplasty (5.4% vs. 29%, *P* = 0.015). Consistent with prior research, this study also identified that intraoperative disruption of the C7 spinous process insertion point is a risk factor for the occurrence of AS following lumbar puncture. Therefore, it is essential to protect the integrity of the C7 spinous process insertion point during surgery.

Another risk factor for AS development is damage to the facet joint. Prior research indicates that detachment, necrosis, and scarring of the soft tissues around the facet joint are closely associated with postoperative AS, and approximately 40% to 55% of chronic neck pain is related to facet joint issues [29, 30]. This may result from surgical stimulation of the posterior nerve branch nerve receptors, and joint surface damage during surgery could lead to aseptic inflammation, causing persistent neck pain. Hence, both suture and traction of the facet capsule can contribute to the occurrence of AS after lumbar puncture, requiring special attention to maintaining the integrity of the facet joint capsule during the procedure.

Studies have shown that the more intact the preservation of posterior neck muscles and ligaments postoperatively, the lower the incidence of AS [31, 32]. When the preoperative cervical curvature is insufficient, patients often experience discomfort in the neck and shoulder regions, leading to compensatory loss of physiological lordosis in the cervical spine. To maintain sagittal plane mechanical balance, the muscles and ligaments are subject to prolonged contraction and fatigue, leading to atrophy and degeneration, ultimately resulting in axial symptoms such as neck and back pain. Therefore, a larger preoperative cervical curvature serves as a protective factor against AS development, and when the preoperative cervical curvature is less than 16.5°, there is a higher risk of AS postoperatively. Moreover, substantial loss of cervical curvature is also a risk factor for AS occurrence since significant and rapid loss of curvature can lead to local structural disruptions, causing localized symptoms due to poor adaptation. Furthermore, a significant decrease in cervical mobility is also a risk factor for AS development. Previous research has indicated that the loss of cervical mobility may not be the cause but rather a consequence of axial pain, where axial pain restricts cervical movement [33]. However, this study administered X-ray and CT examinations to AS patients after taking nonsteroidal anti-inflammatory drugs (Gabapentin 0.3 g tid po) for temporary pain relief and found that the degree of cervical mobility loss in Group A remained higher than in the non-AS group. The difference between the two groups was statistically significant, leading to the belief that a significant loss of cervical mobility is a risk factor for AS development.

Anatomical research has shown that due to the unique anatomical structure of the cervical spine, it is challenging to directly address compression on the dura mater anteriorly during lumbar puncture surgery, relying solely on the “bowstring” principle for indirect decompression [34]. The degree of laminectomy determines the space available for posterior spinal cord drift. A larger laminectomy angle provides more drift space and better recovery of neurological function. However, a larger laminectomy angle also increases the asymmetry of the cervical posterior bony structures, resulting in increased traction on the small joint capsules. Additionally, it can cause separation of paraspinal muscles from the lamina, leading to muscle atrophy. Furthermore, a larger laminectomy angle leads to increased posterior spinal cord drift distance, resulting in nerve root traction within the osseous fibrous canal, causing axial pain. Therefore, the laminectomy angle is considered a risk factor for AS development.

Finally, we found that early postoperative functional exercises are another protective factor against AS occurrence. Early scholars commonly believed that postoperative stability in the cervical spine was poor and discouraged early neck muscle exercises for patients [35]. However, clinical observations have shown that early active flexion–extension exercises of the neck after surgery effectively prevent atrophy of the posterior neck muscles and excessive scar tissue proliferation, significantly reducing the occurrence of stiffness and pain in the neck postoperatively [36, 37]. This study suggests that early postoperative functional exercises significantly reduce the incidence of AS. Therefore, it is necessary to perform early neck muscle exercises based on each patient's condition, especially for those with good wound healing and no significant complications, starting two weeks post-surgery.

### Shortcomings

However, this study has several limitations that need to be acknowledged. Firstly, it is a single-center retrospective study, and though we have attempted to include a sufficient number of risk factors, there might still be some potential risk factors that were not considered, for instance, the BMI, the degree of the disk degeneration before the surgery, etc. In addition, while the study has identified the risk factors for AS development, some of these factors have not been further quantified, such as the specific degree of laminectomy angle. Furthermore, the obtained ROC-AUC values for certain critical cutoff points of risk factors in this study are still relatively low, casting doubt on the practical feasibility of their clinical application. Lastly, the follow-up duration for some patients was relatively short, and more extended, long-term follow-up is necessary to ascertain specific outcomes. Therefore, future research should focus on conducting multi-center studies with larger sample sizes and longer follow-up periods to further validate and expand upon our findings.

## Conclusion

The incidence of AS after lumbar puncture was found to be 40.2%. High preoperative anterior encroachment on the spinal canal, intraoperative facet joint damage, disruption of the C7 spinous process insertion point, and increased laminectomy angle were identified as significant risk factors for AS development. Additionally, greater loss of postoperative cervical curvature and reduced cervical mobility were associated with an increased likelihood of AS occurrence. On the other hand, a larger preoperative cervical curvature and early postoperative functional exercises showed potential as protective factors against AS development.

## Data Availability

The datasets used and/or analyzed during the current study available from the corresponding author on reasonable request.
